# WiN-Reha—effectiveness and durability of effects of orthopedic rehabilitation programs and the study of psychological determinants of aftercare behaviors: a study protocol

**DOI:** 10.3389/fresc.2024.1333924

**Published:** 2024-03-12

**Authors:** Katharina Feil, Julian Fritsch, Susanne Weyland, Lena-Marie Rittmann, Detlef Schmidt, Darko Jekauc

**Affiliations:** ^1^ Institute of Sports and Sports Science, Karlsruhe Institute of Technology, Karlsruhe, Germany; ^2^ Deutsche Rentenversicherung Knappschaft-Bahn-See, Bochum, Germany

**Keywords:** rehabilitation, physical activity, effectivity, long-term, health, determinants

## Abstract

**Background and aim:**

Rehabilitation programs have been shown to have a positive impact on patients' health and work ability. However, the durability of these effects and the extent to which patients alter their health behaviors remain underexplored. This study is divided into two parts; the first assesses the effectivity of rehabilitation programs for orthopedic patients and the durability of effects. The second part examines psychological determinants of aftercare health behaviors.

**Subject and methods:**

Study Part 1 employs a longitudinal study design with up to nine measurement occasions encompassing a three-year follow-up period. Treatment is provided as per orthopedic indications through rehabilitation centers. Measures include subjective health, work ability, body weight, and physical activity behavior. Study Part 2 incorporates a mixed-methods design, involving both quantitative and qualitative assessments. The quantitative component aims to recruit a subsample from Study Part 1 to assess psychological determinants of aftercare health behaviors over 12 to 24 weeks using Ambulatory Assessment. The qualitative component aims to explore the reasons for maintenance and discontinuation of health behaviors and involves a reflexive thematic analysis of interviews with at least 16 individuals, analyzing the differences between those who adopt and those who discontinue their aftercare health behavior.

**Discussion:**

This comprehensive research project may offer insights into the long-term effectivity of rehabilitation programs. Furthermore, it may foster a more profound understanding of the successful incorporation of health-promoting aftercare behaviors, such as physical activity, into everyday life. Therefore, this study may contribute significantly to the evolving field of patient-centered rehabilitation.

**Trial registration:**

The trial has been registered at the German Register of Clinical Studies (DRKS) with the registration number: DRKS00032257

## 1 Background

Rehabilitation services are a vital and integral component of the German health care system, especially in the context of orthopedic impairments. These services not only constitute a significant financial investment within the system (1, 2) but also play a crucial role in enabling individuals with health impairments to return to work and regain quality of life. On the one hand, patients are supported in their healing process by health professionals, aiming to enhance their overall health and work ability as they progress through the program. On the other hand, patients are educated and guided to adopt health-promoting behaviors, such as regular physical activity, to promote long-term health. This dual focus ensures that patients not only recover but also acquire the necessary skills and understanding to maintain their health in the long-term, reducing the likelihood of requiring further rehabilitation services. By targeting these areas, rehabilitation programs are designed to provide comprehensive care that addresses both the immediate needs and future well-being of patients with orthopedic impairments.

Studies on cardiovascular (3), oncological (4), neurological (5) and musculoskeletal (6, 7) rehabilitation interventions showed positive effects on previously impaired health parameters. In addition, a reduced mortality rate was observed (3, 8). A recent meta-analysis yielded that orthopedic rehabilitation programs in Germany increase patients' quality of life and work ability (9). However, the existing research on the effectiveness of rehabilitation programs reveals some critical gaps and shortcomings that warrant further investigation. Most notably, a control group is an essential component for robustly evaluating the effectivity of the intervention which is missing in many studies. Moreover, the durability of these positive effects remains largely unclear, with few studies incorporating a follow-up period exceeding 12 months (9). This research gap regarding long-term durability underscores the need for more comprehensive research to optimize the effectivity of rehabilitation programs.

Physical activity is a critical aspect of rehabilitation, particularly for maintaining benefits after rehabilitation. In addition, it reduces the likelihood of overweight and obesity (10–12). However, understanding the underlying psychological processes that influence the adoption and maintenance of aftercare health behaviors such as physical activity is complex. Dual process approaches suggest that two processes, one that is automatic and implicit and another that is reflective and explicit, influence health behavior (13, 14). For example, in the Physical Activity Adoption and Maintenance Model [PAAM, (14)], the variables affect and habit are rather described as implicit processes, while intention is rather understood as a construct of explicit processes. The theory postulates that these variables are influenced on the one hand by the experience during physical activity and on the other hand also influence future activity behavior. Current research indicates that especially intention (15), perceived behavioral control (16), anticipated affect (17), remembered affect (18), habit (19) and intrinsic motivation (20) may explain health behavior.

### 1.1 Objectives of the study

WiN-Reha (“Wirksamkeit und Nachhaltigkeit von Rehabilitationsmaßnahmen und die Analyse von Determinanten des Gesundheitsverhaltens”) is the acronym for the project and comprises two study parts, each with specific objectives aimed at contributing to the development of rehabilitation programs for orthopedic impairments. The study takes a comprehensive approach by employing both longitudinal and mixed-methods designs to investigate various aspects of rehabilitation.

#### 1.1.1 Study Part 1: effectiveness and durability of effects of orthopedic rehabilitation programs

The primary objective of Study Part 1 is to evaluate the effectiveness of rehabilitation programs for patients with orthopedic impairments. The focus extends beyond the immediate outcomes, aiming to investigate the durability of effects. The criteria for evaluating the effectiveness and durability are: (i) subjective physical and mental health, (ii) work ability, (iii) body weight, and (iv) physical activity. Subjective health and work ability are crucial indicators to return to work which is the main aim of rehabilitation providers. Close correlates of these indicators and a healthy lifestyle are participants' body weight and the amount of regular physical activity in everyday life.

#### 1.1.2 Study Part 2: study of psychological determinants of aftercare health behaviors

Study Part 2 adopts a mixed-methods design consisting of quantitative and qualitative methods. The objective of the quantitative component is to analyze the influence of specific psychological determinants on aftercare health behaviors. This includes examining how intention, anticipated affect, experienced affect, habit, and motivation influence the adoption and maintenance of aftercare health behaviors. The qualitative study aims to analyze the conditions that affect success or failure in completing an aftercare health behavior. By exploring patients' experiences and challenges, this part of the study seeks to gain knowledge about why patients either follow through with their aftercare health behavior or drop out.

## 2 Methods

The methods of Study Part 1 and Study Part 2 will be explained in separate sections.

### 2.1 Methods of Study Part 1

#### 2.1.1 Sampling and participants

Eligibility criteria for participating in the study are that patients need to be at least 18 years old, have basic knowledge of the German language, and the reason for their rehabilitation is an orthopedic impairment prescribed by the German pension fund. There are no further inclusion criteria regarding the type of orthopedic impairment. Participants will be recruited at two different measurement occasions, one before the rehabilitation program starts and one during the first few days of the rehabilitation program. For the first measurement occasion, patients receive information about the study and the first questionnaire via post several weeks before the rehabilitation program starts (t_0_). Rehabilitation centers send out the study material together with the usual information material regarding the rehabilitation program itself. Patients that did not participate in the study at the first measurement occasion can still be recruited at the second measurement occasion (t_1_) which is taking place at the beginning of the rehabilitation program in the rehabilitation centers. Participants that already participated in the first measurement occasion (before rehabilitation started) also fill out the second questionnaire at the beginning of the rehabilitation program (t_1_). This procedure allows for a comparison of the indicators' development from the time without a rehabilitation program with the indicators' development after the rehabilitation program has started. Participants will be informed of the purpose of the study and will give their written consent to participate voluntarily. Currently, five rehabilitation centers in Germany are part of the study. The patients will receive a 50€ voucher, if they fill out the questionnaires at all measurement occasions. Participants are reminded by the research team via e-Mail or phone to fill out the questionnaires to increase participation in the study.

A power analysis was conducted with a small effect size as the evidence regarding the efficacy of rehabilitation programs is insufficient. A meta-analysis about the effects of cardio-vascular rehabilitation on health-related quality of life yielded effect sizes of 0.17–0.25 (21). Due to a large heterogeneity in this meta-analysis and because cardio-vascular rehabilitation programs are different from orthopedic rehabilitation programs, a standard mean effect size of 0.10 was chosen. The power analysis with Cohens *f* = 0.05, *α *= 0.05, 1-*β* = 0.80, a correlation among repeated measures of *r* = 0.50 and a design with eight measurement occasions since the start of rehabilitation program using an analysis of variance (ANOVA) with repeated measurements (within-between interaction) resulted in *n* = 360 participants. Considering a dropout after each measurement occasion of 15%, the recruitment of *n* = 1,101 participants is planned.

#### 2.1.2 Rehabilitation programs

Orthopedic rehabilitations programs in Germany are offered stationary or as an outpatient program in rehabilitation centers. Typically, rehabilitation programs last three to four weeks and comprise different services such as physiotherapy, medical training therapy, and health education. For some indications, standards for treatments exist based on scientific evidence such as for knee and hip joints, but this is not the case for all orthopedic indications (22). Therefore, the content and structure of rehabilitation programs can vary between rehabilitation centers. Rehabilitation programs at the participating rehabilitation centers will be executed as usual and the impact of the study will be limited to the data collection. Therefore, this treatment is not an intervention provided by the research team, but rather treatment as usual.

#### 2.1.3 Data collection

**Figure 1:**
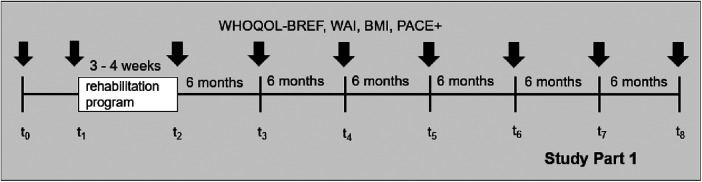
Study design of Study Part 1.

Data collection started in September 2023 and baseline measurements will be completed approximately in January 2025. The study design consists of nine measurement occasions for participants that are recruited in the time before the rehabilitation program starts (t_0_–t_8_). For participants that are recruited at the beginning of the rehabilitation program in the rehabilitation center, the study comprises eight measurement occasions (t_1_–t_8_). Pre (t_1_) and post (t_2_) measurements are conducted at the beginning and at the end of the rehabilitation program to assess the effectivity. Based on their preference, the participants are contacted by e-mail or mail for the further measurement occasions (t_2_–t_8_). The interval between the measurement occasions after the rehabilitation is completed (t_2_–t_8_) is six months, resulting in a total follow-up period of three years. The study design for Study Part 1 is provided in Figure 1.

Demographic information, including age, gender, nationality, and current employment situation, are asked at enrolment. The participants are also asked to provide contact details for follow-up measurement occasions. Post measurement (t_2_) comprises also information about the rehabilitation program and health indicators for subgroup-analyses. Information regarding the employment status and disability pension are reported to the research team of the Karlsruhe Institute of Technology (KIT) via the Deutsche Rentenversicherung (DRV) Knappschaft-Bahn-See during the last year of follow-up, if participants gave their consent at t_0_ or t_1_. All other instruments described below are used at the baseline measurement (t_0_), the pre and post measurements (t_1_, t_2_), and the follow-up measurements (t_3_–t_8_).

##### 2.1.3.1 Health-related quality of life

A commonly used measure of health-related quality of life is the World Health Organization Quality-of-Life Scale (WHOQOL-BREF) questionnaire developed by the World Health Organization (23). This questionnaire contains 26 items with the dimensions physical health, mental health, social relationships and environment. In this study the scales physical health and mental health with a total of 13 items are used. A large-scale study conducted in 23 countries showed good reliability for the physical health (*α* = 0.82) and mental health (*α* = 0.81) scales (24). The study also supported the construct validity of the questionnaire.

##### 2.1.3.2 Work ability

The Work Ability Index (WAI) is an internationally established instrument for measuring work ability (25). The short version consists of ten questions covering seven dimensions: (1) current work ability compared to the best ever, (2) current work ability compared to the demands of the job, (3) number of current illnesses diagnosed by a doctor, (4) estimated impairment of work performance due to illness, (5) days of sick leave in the last 12 months, (6) assessment of one's own work ability in two years, and (7) mental performance reserves. The result is a total score between 7 and a maximum of 49, which indicates the assessed ability to cope with existing work demands (25). The reliability of the WAI can be considered satisfactory with a Cronbach's alpha of 0.78. With regard to the validity of the questionnaire, the WAI has been shown to be able to predict early retirement and the duration of long-term disability (26, 27).

##### 2.1.3.3 Weight status

The Body Mass Index (BMI) is a widely used measure of overweight and obesity. According to a classification provided by the World Health Organization (WHO), a person is considered overweight with a BMI of 25–30 kg/m^2^ and obese with a BMI of ≥30 kg/m^2^. Obesity is also classified according to severity (28). Thus, adults with a BMI of 30–34.9 kg/m^2^ are classified as severity I, adults with a BMI of 35–39.9 kg/m^2^ as severity II, and adults with a BMI of ≥40 kg/m^2^ as severity III. The BMI is considered an appropriate measure of overweight alongside other measures such as waist circumference or waist-hip ratio (29, 30). Participants are asked to provide information about their body weight in kilograms and height in meters.

##### 2.1.3.4 Physical activity

The items of the Patient-Centered Assessment and Counselling for Exercise Plus Nutrition (PACE+), which were originally developed for adolescents (31), are used to assess physical activity in everyday life. The items were translated into German (32) and successfully used in comprehensive long-term studies not only with children and adolescents, but also with adults (33, 34). Two items record how many days per week participants were active for at least 60 min at moderate intensity. The first item refers to the last week and the second to a normal week. The response scale ranges from 0 to 7 days. Before answering these questions, physical activity is defined as any activity that makes the heart beat faster and increases breathing for some time. Examples such as walking to work, running, dancing, and swimming are given. The average of the two items builds the final physical activity score. The items showed interclass correlation coefficients between 0.64 and 0.79, which can be considered good. Validity can also be rated as good, as data from the PACE + items correlated significantly with accelerometer data [*r* = 0.40, *p* < .001, (31)]. A study using the German formulation of the items showed comparable values in terms of test retest reliability (*r* = 0.68, *p* < 0.05) and validity (*r* = 0.29 with accelerometer data, *p* < 0.05) (33).

##### 2.1.3.5 Employment status

Employment status of the participants are provided by the pension fund once a year during follow-up (three measurement occasions in total), if the participants gave their consent at t_0_ or t_1_. The data reflect, the number of days the participants were employed between the measurement occasions. If participants were not employed, information are provided on the payment of compensation for these periods (e.g., unemployment benefit, disability pension, care support allowance).

#### 2.1.4 Data analysis

Data will be analyzed by the KIT research team. Questionnaire data are pseudonymized through personal codes and contact details are stored separately from questionnaire data. The identification of participants is possible only through a password-protected, locally stored file by the research team. A research assistant independently checks the accuracy of the data entry. Missing data will be analyzed to what extent they are systematic and, if necessary, treated with multiple imputation or the full-information maximum likelihood approach.

In order to ensure comprehensive analysis and interpretation of the data collected in our study, a multi-faceted statistical approach will be employed. Initially, repeated measures analyses of variance will be utilized to assess changes in subjective health, work ability, BMI, and physical activity behavior over time. This method is particularly effective for analyzing data where multiple measurements are recorded for the same subjects. Analysis of variance with repeated measurement allows for the evaluation of both within-subjects effects (changes over time) and between-subjects effects (differences among groups). In addition, latent growth curve modeling (LGCM) will be implemented to explore the trajectories of the aforementioned parameters across the study period. LGCM provides a flexible framework to model nonlinear growth trajectories, accommodating various shapes of developmental change (35). This is particularly pertinent for our study, as it enables the examination of individual differences in change patterns, thereby offering a deeper understanding of how rehabilitation programs impact individuals over time. Age, gender, the duration and the completement of the rehabilitation program will be included as control variables.

### 2.2 Methods of Study Part 2

#### 2.2.1 Sampling and participants

Participants of Study Part 1 are eligible for Study Part 2, if they plan to implement an aftercare health behavior on a regular basis after their rehabilitation program ends (e.g., participation in an after-care program). The data collection for this study part has not started yet. Researchers will contact participants that gave their written consent to participate in Study Part 2 at t_0_ or t_1_. Participants will inform the researchers about their planned aftercare health behavior (i.e., the specific days during the week and the time planned to execute the behavior). In the quantitative part of Study Part 2, psychological determinants of the aftercare health behavior will be assessed using Ambulatory Assessment. Researchers will send short questionnaires via the application movisensXS to the participants on different measurement occasions during the week dependent on the selected days and times of the aftercare health behavior. Participants will also be asked whether they performed the planned behavior and to state the reasons if not. Missing planned physical activity sessions are not a reason for dropout, as long as the participant will plan to continue with the health behavior. In the qualitative part of Study Part 2, participants of the quantitative part will be interviewed, which either successfully implemented the aftercare health behavior or failed to implement it on a regular basis. Both groups will be interviewed to gather more information about the reasons that contributed to their success or failure.

As no treatment is planned for Study Part 2, a small effect size is assumed. For sample size estimation with G*Power, the more conservative repeated measures ANOVA is used, because power analysis for multilevel models requires assumptions about several population parameters that were not found in the literature. Estimating the effect size with Cohen's *f* = 0.10, *α* = 0.05, 1-*β* = 0.80, a correlation between the repeated measures of *r* = 0.50 and 12 repeated measures in a repeated measures ANOVA (interaction of within-subject and between-subject effects) resulted in a total sample size of 72 participants. Assuming that 10% of participants drop out of the study each week over a 12-week study period (36), 226 subjects need to be recruited. A purposive sampling (37) will be used to interview (A) people who have integrated the aftercare health behavior into their everyday life and (B) people who have stopped the aftercare health behavior. In each group, a minimum of eight people will be interviewed to gather different perspectives on the topic.

#### 2.2.2 Aftercare health behavior—Study Part 2

Participants can choose a health promoting behavior with the main focus on physical activity. This may be an offered aftercare program such as IRENA (Intensified Rehabilitation Aftercare) provided by certified rehabilitation centers or another aftercare health behavior with the goal of stabilizing the effects gained during the rehabilitation program. There is no intervention provided by the research team or the rehabilitation center where the participants completed their rehabilitation program.

#### 2.2.3 Data collection

**Figure 2:**
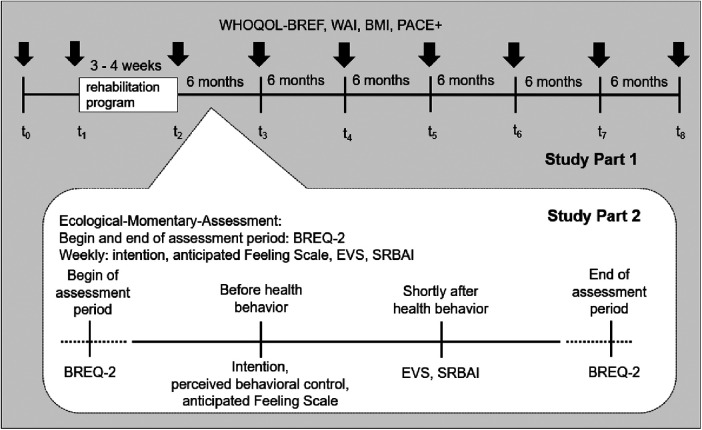
Study design of Study Part 2.

Data collection of Study Part 2 will start in February 2023 and measurements will be completed approximately in July 2025. Study part 2 begins as soon as the participant takes up the aftercare health behavior after rehabilitation. For each week we will assess the engagement in the aftercare health behavior. The measurement occasions of the psychological variables will depend on the days and time of the participant's aftercare health behavior. While intention and anticipated affect will be assessed before the aftercare health behavior, remembered affect and habit will be measured after the aftercare health behavior. Motivation will be assessed at the beginning of the assessment period of Study Part 2 and at the end. Questionnaires will be sent via the application MovisensXS to participants' smartphones. The duration of Study Part 2 depends on the chosen aftercare health behavior and on the participants' willingness to maintain it. A duration of 12 weeks is currently scheduled with the option to extend the duration up to 24 weeks (e.g., IRENA lasts 24 weeks). Figure 2 shows the measurement occasions dependent on the aftercare health behavior.

##### 2.2.3.1 Intention

Intention is a complex construct with two components: decisional intention reflects the direction of the intention (toward aftercare health behavior or toward an alternative behavior), while intention strength represents the commitment to enact behavior in that direction [intensity of intention, (38)]. Decisional intention to perform the aftercare health behavior will be assessed with the item “Do you intend to do … (specific behavior)” and can be answered with “yes” or “no”. If “yes”, intention strength will be measured with the item “To what extent do you intend to do … (specific behavior)”, and can be answered on a seven-point response scale ranging from 1 (very low) to 7 [very high, (38)].

##### 2.2.3.2 Perceived behavioral control

Perceived behavioral control will be assessed with one item measuring the level of ones perceived control related to showing the aftercare behavior: “It is within my control to participate in … (e.g., the IRENA session).” Participants will respond to a Likert scale ranging from 1 (strongly disagree) to 7 (strongly agree). Similar items have been part of previous studies measuring perceived behavioral control to predict health behavior showing good reliability (39, 40).

##### 2.2.3.3 Anticipated affect

An adapted version of the Feeling Scale is used to measure anticipated affect. The Feeling Scale is a well-established single-item measure of affective valence (41). The adapted version of the Feeling Scale asks participants about their expectations of how they will feel during the aftercare health behavior. The response scale ranges from −5 (very bad) to +5 (very good). Similar versions of the Feeling Scale have already been used in previous studies (42, 43).

##### 2.2.3.4 Remembered affect

Remembered affect will be measured shortly after the aftercare health behavior with the Empirical Valence Scale (EVS). The EVS consists of a single item to measure how well participants felt while performing a health behavior (44). The visual analogue scale ranges from −100 (most unpleasant imaginable) to +100 (most pleasant imaginable) and thirteen verbal anchors (e.g., strongly, barely, slightly) are placed throughout the scale. The EVS has been used in previous studies to measure remembered affect (45).

##### 2.2.3.5 Habit

Habit will be assessed after the aftercare health behavior was shown with one item:

“Attending the … (e.g., today's IRENA session) was something I did automatically.” The response scale consists of four items on a five-point Likert scale ranging from 1 (strongly disagree) to 5 (strongly agree). The item is based on the Self-Report Behavioral Automaticity Index (SRBAI) and measures the automaticity of the behavior as a central feature of habit (46) and has been used in a previous study (47).

##### 2.2.3.6 Motivation

Motivation is assessed using the German version of the Behavioral Regulation Exercise Questionnaire-2 (BREQ-2), which consists of 19 items with a 5-point response format (48). It covers five different types of motivation: (1) amotivation, (2) external regulation, (3) introjected regulation, (4) identified regulation, and (5) intrinsic motivation. In addition to the individual manifestations of motivation, the Relative Autonomy Index (RAI) can also be calculated, which represents a person's degree of self-determination in relation to a behavior (48). Cronbach's alpha is 0.60 for amotivation, 0.77 for external regulation, 0.77 for introjected regulation, 0.83 for identified regulation, and 0.88 for intrinsic regulation (49). In terms of validity, the subscales were found to correlate with each other consistent with the theory, and higher self-determined motivation was associated with an increased likelihood of engaging in health behavior (49).

#### 2.2.4 Data analysis

In order to quantify the effects of psychological determinants on aftercare health behaviors, multilevel models will be calculated. The advantage of multilevel models in the analysis of repeated measures data is their flexibility in dealing with unbalanced data structures, nested samples (e.g., clustering in rehabilitation centers), and missing values (50). This modeling technique is particularly suited for analyzing data that is hierarchically structured, nested in treatment centers or involves repeated measures, thus allowing for the assessment of both individual and group-level variations (e.g., analysis of the influence of programs in different rehabilitation centers). In addition, latent growth curve models will be used to examine the relationships between the growth parameters of intentions, anticipated affect, experienced affect, habit, and aftercare health behaviors. This modeling approach facilitates an in-depth exploration of individual growth trajectories and their interrelations over time, offering valuable insights into the developmental aspects of health behaviors post-rehabilitation.

The interviews will be analyzed using reflexive thematic analysis. Specifically, the six-phase model (51) will be applied. In order to increase the trustworthiness of the analysis, the interviews will be analyzed independently by two persons. In addition, other researchers will serve as critical friends to contemplate alternative interpretations of the interviews (52). These researchers will provide independent perspectives and assist in exploring alternative interpretations, thereby enhancing the depth and credibility of our qualitative findings.

## 3 Discussion

The aims of this study are (1) to evaluate the effectiveness and the durability of effects of orthopedic rehabilitation programs and (2) to gather a deeper understanding of how psychological determinants influence aftercare health behaviors.

Study Part 1 includes a three-year follow-up period after rehabilitation, which is much longer than most previous studies (9) and enables the evaluation of long-term effects. Additionally, a control period without treatment (i.e., before the rehabilitation program starts) is planned. In Study Part 2, the use of Ambulatory Assessment allows for data collection in daily life that is aligned with the time the participants execute their individual aftercare health behavior. In addition, an advantage of mixed methods designs is that qualitative data help explain results of quantitative data. Therefore, interviews with participants may provide additional information about how psychological determinants interact with the individual aftercare health behavior.

Possible challenges include the recruitment of patients prior to the start of the rehabilitation programs to build an adequate control group. Patients will receive the study material via post and a promotion letter of the rehabilitation center is supposed to increase the recruitment rate prior to rehabilitation. Furthermore, long follow-up periods with many measurement occasions often result in high drop-out rates and sample attrition (53). For that reason, researchers will send out reminders to fill out all questionnaires. Regarding Study Part 2, participants may change the aftercare health behavior they chose and switch time and dates when they plan to execute their behavior.

The importance of this research project lies in its comprehensive examination of two interrelated aspects vital to the field of rehabilitation: the effectiveness and durability of effects of rehabilitation programs (Study Part 1) and the psychological determinants of aftercare health behaviors that contribute to long-term health (Study Part 2). Although orthopedic rehabilitation programs in Germany seem to have positive effects on quality of life and work ability (9), the long-term impact of these treatments have not been evaluated yet. In addition, gaining knowledge about how health behaviors can be transferred into everyday life is of great importance to ensure the success of the rehabilitation. These insights hold significant potential for the optimization of rehabilitation programs, fostering lasting health behaviors after rehabilitation, and enhancing overall patient outcomes. Furthermore, the evidence-based refinements of rehabilitation programs suggested by this research could lead to more efficient resource allocation and reduced costs for the healthcare system. By linking clinical practice to patients' psychological determinants and personal experiences, the study contributes to a more patient-centered and economically sustainable approach for orthopedic rehabilitation.

## Trial status

The data collection of Study Part 1 started in September 2023.

## Ethics statement

The studies involving humans were approved by Karlsruhe Institute of Technology. The studies were conducted in accordance with the local legislation and institutional requirements. The participants provided their written informed consent to participate in this study.
